# Vibriocidal Titer and Protection From Cholera in Children

**DOI:** 10.1093/ofid/ofz057

**Published:** 2019-02-11

**Authors:** Alaina S Ritter, Fahima Chowdhury, Molly F Franke, Rachel L Becker, Taufiqur R Bhuiyan, Ashraful I Khan, Nirod Chandra Saha, Edward T Ryan, Stephen B Calderwood, Regina C LaRocque, Jason B Harris, Firdausi Qadri, Ana A Weil

**Affiliations:** 1 Infectious Diseases Division, Massachusetts General Hospital, Boston; 2 Department of Medicine, Harvard Medical School, Boston, Massachusetts; 3 Department of Global Health and Social Medicine, Harvard Medical School, Boston, Massachusetts; 4 Department of Microbiology and Immunobiology, Harvard Medical School, Boston, Massachusetts; 5 Department of Pediatrics, Harvard Medical School, Boston, Massachusetts; 6 Vaccine Science, International Center for Diarrheal Disease Research, Bangladesh, Dhaka; 7 Department of Immunology and Infectious Diseases, Harvard School of Public Health, Boston, Massachusetts; 8 Division of Global Health, MassGeneral Hospital for Children, Boston, Massachusetts

**Keywords:** cholera, *Vibrio cholerae*, vibriocidal titer

## Abstract

**Background:**

Vibrio cholerae, the causative agent of cholera, is a major cause of diarrhea worldwide. Children under the age of 5 have the highest disease burden of cholera. Vibriocidal antibody responses following natural infection and oral cholera vaccination (OCV) are associated with protective immunity, but whether this holds uniformly true in young children is not known.

**Methods:**

Household contacts of cholera patients are at high risk of *V* cholerae infection. We measured the association between baseline vibriocidal titer and the subsequent risk of infection in 50 household contacts <5 years old, 228 contacts 5–15 years old, and 548 contacts 16–70 years old in Bangladesh to determine whether vibriocidal antibody responses predict protection from *V* cholerae infection equally in all age groups.

**Results:**

We found that the vibriocidal titer predicted protection similarly in young children and other age strata. There was no interaction between age and vibriocidal titer. Mean baseline serum vibriocidal titers were higher in individuals in all age groups who remained uninfected compared with those who developed *V* cholerae infection during the follow-up period.

**Conclusions:**

After OCV, children have comparable vibriocidal responses to adults but a shorter duration and magnitude of protection compared with adults. In persons exposed to natural infection, we found that the vibriocidal titer predicts protection uniformly in all age groups. The vibriocidal titer may not be the optimal marker to demonstrate protection after OCV, and improved markers for estimating OCV efficacy in children are needed.

*Vibrio cholerae* infection causes severe diarrhea resulting in dehydration and is responsible for over 100 000 deaths annually [1]. Two primary serogroups of *V cholerae,* O1 and O139, can cause epidemic disease. The O1 serogroup is further subdivided into the Ogawa and Inaba serotypes.

Infection with *V cholerae* O1 results in protective immunity against future infection [2]. The serum vibriocidal antibody is the most common correlate of immunity, and although increased vibriocidal titers correlate with protection from disease, no threshold level is completely predictive of protection. Vibriocidal antibodies are bactericidal, complement-fixing, and target the O-specific antigen of the lipopolysaccharide [2, 3]. In cholera-endemic areas, elevated baseline vibriocidal titers are positively associated with age, presumably due to recurring natural exposure to *V cholerae* [4]. However, in the immediate convalescent period (up to 30 days) after symptomatic cholera, titers are similar in children 2 to 5 years of age, older children, and adults [4, 5].

Young children, older children, and adults also develop robust and comparable vibriocidal responses to oral cholera vaccination, although responses may be diminished in very young children (under 2 years of age) [6]. However, compared with adults and older children, vaccination is less effective in children under 5 years of age [7, 8]. We questioned whether vibriocidal titers accurately predict protection from infection in children as precisely as in adults. We have previously described the relationship between the vibriocidal titer and protection in 2 cohorts of household contacts of cholera patients without a detailed analysis of all age groups, due to limited numbers of young children enrolled [4, 9]. Therefore, to achieve larger numbers of children, we added a recent cohort of household contacts at high risk of *V cholerae* infection to 2 previous cohorts to determine whether protection was uniformly associated with a higher baseline vibriocidal antibody titer across the age spectra.

## MATERIALS AND METHODS

### Subject Enrollment and Clinical Outcomes

Household contacts of cholera patients in Dhaka city were enrolled at the International Centre for Diarrheal Disease Research, Bangladesh (icddr,b). Household contacts are at high risk for infection, and approximately 20% of contacts become infected with *V cholerae* in the week after a household member is hospitalized with cholera [10]. In this analysis, 3 cohorts of household contacts were studied. The first was enrolled between 2001 and 2005. Index cases with acute watery diarrhea presented to the icddr,b, and after a confirmatory *V cholerae* stool culture, household contacts without severe comorbid conditions were enrolled in the study. Blood samples for blood group determination and vibriocidal antibody titers were drawn upon enrollment (day 2) and on study days 4 and 21. On study days 2–7 and 14, trained study field workers visited contacts at home to obtain rectal swabs and a symptom history, and contacts were seen at the center on day 21. The second and third cohorts of contacts were enrolled from 2006 to 2011 and 2012 to 2017, respectively, and blood samples were drawn on days 2, 7, and 30. Rectal swabs and symptom histories in these latter 2 cohorts were collected for a shorter duration of 9 days (study days 2 through 10) after results from the first cohort indicated that household contacts were most likely to develop infection within days of the household case hospitalization [10]. Household contacts were defined as infected with *V cholerae* if they had a culture positive rectal swab for *V cholerae* at any point during the follow-up period. Data from the 2 previously described cohorts were combined with the 2012–2017 cohort to enable evaluation of the relationship between vibriocidal titer and protection in all ages, including young children. The Institutional Review Board of the Massachusetts General Hospital and the Research and Ethical Review Committees of the icddr,b approved this human study. All participants or their guardians gave written informed consent.

### Vibriocidal Antibody Assay

Baseline plasma vibriocidal titers were measured using guinea pig complement and the homologous O1 Ogawa and O1 Inaba serotypes as described previously [11]. The titer recorded was the reciprocal of the highest dilution that reduced the optical density by >50% compared with the controls.

### Statistical Analysis

The outcome of interest was infection with *V cholerae* (defined as a positive rectal swab culture) at any time during follow-up. We compared the mean log vibriocidal titers among those who remained uninfected and those who became infected with *V cholerae* during follow-up. We stratified by age group (<5 years, 5–15 years, 16–70 years), and the 9- to 21-day occurrence of infection was stratified by titer (≤20, 40–80, and ≥160) and age group. To test for a relationship between log vibriocidal titer and *V cholerae* infection in each age group, we generated a binomial regression model for each age group. We used generalized estimating equations with a compound symmetry correlation structure to adjust for clustering by household. We considered potential confounding by blood group O status, a host characteristic known to influence susceptibility to *V cholerae* infection [9].

To test whether the association between log vibriocidal titer and *V cholerae* infection differed by age (ie, interaction), we repeated the binomial regression analyses described above among all ages, adjusted for age, and added an interaction term between log vibriocidal titer and age. For all regression analyses, we modeled age as a continuous variable because a goodness-of-fit evaluation using Quasilikelihood under the Independence model Criterion (QIC) suggested that age categories did not improve the fit of the data. This was true for models with and without an interaction term between age and titer. We conducted sensitivity analyses in which we repeated above analyses modeling vibriocidal titer as categorical (≤20, 40–80, and ≥160) and considering only symptomatic *V cholerae* infection. Statistical analyses were performed using Stata/SE 15.1 (StataCorp LLC, College Station, TX), SAS version 9.4 (SAS, Cary, NC), and GraphPad Prism 7.04 (GraphPad Software, La Jolla, CA).

## RESULTS

### Study Population

We enrolled a total of 1741 household contacts. To ensure we compared contacts who developed infection during the follow-up period to those who did not, we excluded contacts with a positive rectal swab at the time of enrollment in the study (121 contacts) or report of vomiting or diarrhea on the day of enrollment or the week prior (240 contacts). We also excluded contacts with an indeterminate clinical outcome during the follow-up period (374 contacts with diarrhea and negative rectal swab cultures for *V cholerae*). Contacts were excluded if they developed a different serotype or serogroup of *V cholerae* infection than the index case in their household (52 contacts), had missing rectal swab data on the day of enrollment (10), were missing symptom information for either the day of enrollment or 1 full week of follow up (22), or were missing a baseline vibriocidal titer or had fewer than 1 follow-up titer recorded (120). Twenty-four contacts were excluded for meeting 2 or more of the above-listed criteria. Our final cohort for analysis was 826 household contacts.

Fifty contacts were <5 years (“young children”), 228 contacts were between 5 and 15 years (“older children”), and 548 contacts were 16–70 years old (“adults”). The median age of contacts was 25 years (range, <1–71). Women (407, 49%) and men participated equally in the study. A total of 153 (23%) contacts became infected with *V cholerae* O1 during follow up, and 673 remained uninfected. Of the infected contacts, 110 (72%) were infected with serotype Ogawa, the dominant serotype at the time, and 43 (28%) were infected with serotype Inaba. Sixty-four contacts were symptomatic (58% of infected contacts).

### Vibriocidal Titers

**Figure 1.**
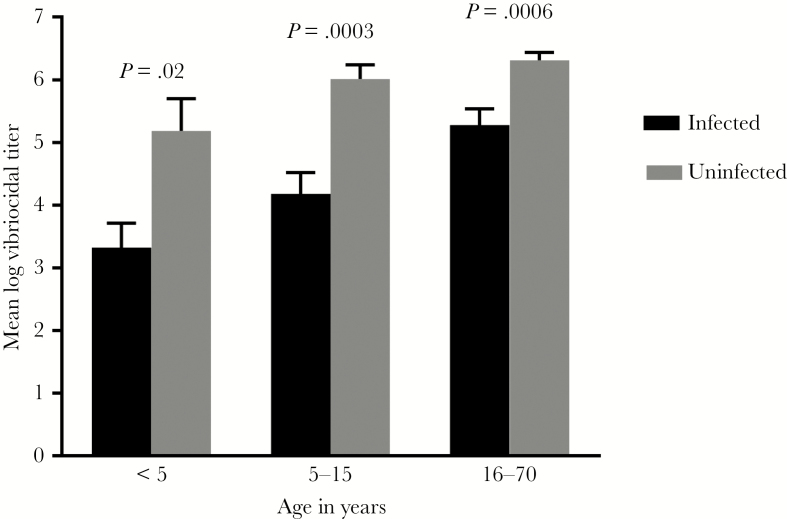
Baseline vibriocidal antibody responses in household contacts of cholera patients. Mean baseline log vibriocidal titers in contacts comparing those who went on to develop infection during the follow-up period and those who remained uninfected. Error bars represent the standard error. For infected contacts, n = 13 for <5, n = 50 for 5–15, and n = 90 for 16–70. For uninfected contacts, n = 37 for <5, n = 178 for 5–15, and n = 458 for 16–70.

**Figure 2.**
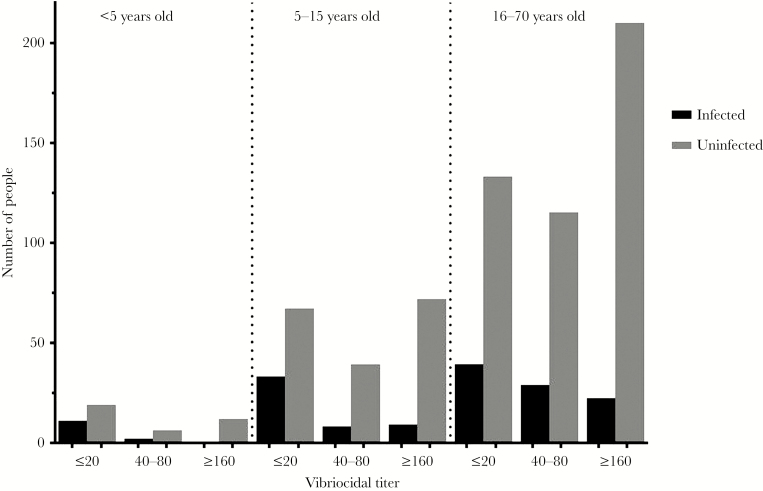
Number of household contacts infected with *Vibrio cholerae* versus uninfected during follow up, stratified by vibriocidal titer. The number of contacts in each group is displayed in Table 1 below.

The mean baseline serum vibriocidal titer was higher in household contacts who remained uninfected in all age groups compared with contacts who developed *V cholerae* infection during the follow-up period (young children, *P* = .02; older children, *P* = .0003; adults, *P* = .0006) (Figure 1). The number of *V cholerae* infections in groups characterized by age and baseline titer are shown in Table 1 and Figure 2.

**Table 1.** Number of *Vibrio cholerae* Infections Among Household Contacts by Age and Baseline Titer^a^

	Day 2 Titer ≤20			Day 2 Titer 40–80			Day 2 Titer ≥160		
Age (Years)	N	N Infected	Percent Infected (95% CI)	N	N Infected	Percent Infected (95% CI)	N	N Infected	Percent Infected (95% CI)
<5	30	11	36.7 (21.9–54.5)	8	2	25.0 (3.2–65.1)	12	0	0.0 (0.0–26.5)^b^
5–15	100	33	33.0 (24.6–42.7)	47	8	17.0 (8.9–30.1)	81	9	11.1 (6.0–19.8)
≥16	172	39	22.7 (17.1–29.5)	144	29	20.1 (14.4–27.4)	232	22	9.5 (6.3–13.9)

Abbreviations: CI, confidence interval.

^a^Contacts were followed for 9 days (337 contacts) to 21 days (489 contacts) from enrollment for rectal swab data and for 21 days (489 contacts) to 30 days (337 contacts) for serum samples, depending on the cohort. Almost all contacts that developed *V cholerae* infection were diagnosed ≤10 days after enrollment (148 of 158; 97%). Confidence intervals represent the expected range if the study population was repeatedly resampled.

^b^Exact CI calculated using exact methods given small numbers.

Adjustment for blood group status did not alter effect estimates and therefore was not included in the final model. Adjusting for age, each unit increase in log titer was associated with a 14% lower risk of infection during follow-up (risk ratio [RR], 0.86; 95% confidence interval [CI], 0.81–0.91), and we found no evidence of interaction between age and vibriocidal titer (*P* value for interaction: 0.80). Results of sensitivity analyses were consistent with the primary analyses. When we modeled vibriocidal titer in categories, we found that relative to a titer <20, a titer of 40–80 was associated with a 24% lower risk of infection (RR, 0.76; 95% CI, 0.53–1.08) and a titer ≥160 was associated with a 63% lower risk of infection (RR, 0.37; 95% CI, 0.25–0.56). These relative risks did not significantly differ by age (*P* for interaction = .96 and .62, respectively). When we restricted the outcome to symptomatic cases of *V cholerae* infection (Table 2), we found that each unit increase in log vibriocidal titer was associated with protection from symptomatic infection (RR, 0.82; 95% CI, 0.73–0.91); however, we found no evidence that this relationship varied by age group (*P* for interaction = .64).

**Table 2.** Occurrence of Symptomatic *Vibrio cholerae* Infection Among Household Contacts by Age and Baseline Titer^a^

	Day 2 Titer ≤20			Day 2 Titer 40–80			Day 2 Titer ≥160		
Age (Years)	N	N Symptomatic	Percent Symptomatic (95% CI)	N	N Symptomatic	Percent Symptomatic (95% CI)	N	N Symptomatic	Percent Symptomatic (95% CI)
<5	30	6	20.0 (7.7–38.6)^b^	8	1	12.5 (0.3–52.7)^b^	12	0	0.0 (0.0–26.5)^b^
5–15	100	16	16.0 (10.1–24.4)	47	3	6.4 (1.3–17.5)^b^	81	3	3.7 (0.8–10.4)^b^
≥16	172	19	11.0 (7.2–16.6)	144	10	6.9 (3.8–12.3)	232	6	2.6 (1.2–5.5)

Abbreviations: CI, confidence interval.

^a^Confidence intervals represent the expected range if the study population was repeatedly resampled.

^b^Exact CI calculated using exact methods given small numbers.

## DISCUSSION

Children younger than 5 years of age are disproportionately affected by cholera, and age is correlated with susceptibility to disease independent of other risk factors [9]. Two cholera vaccines are preapproved for use in both adults and children by the World Health Organization. The first is a whole cell killed vaccine with an added recombinant cholera toxin B subunit ([WC/rBS] Dukoral; Valneva). A precursor of this vaccine was studied in a large randomized trial in rural Bangladesh and demonstrated only 26% protection at 3 years for children vaccinated between ages 2 and 5, with no protection at 5 years [7, 12]. The second, a bivalent killed whole cell vaccine without the B subunit of cholera toxin ([BivWC] Shanchol [ShanthaBiotechnic] and Euvichol [EuBiologics]), includes serogroup O1 and O139 strains. A randomized trial of a 2-dose regimen in a cholera-endemic area demonstrated a 60% reduction in cholera in adults followed for 5 years, with much less protection in young children, and less than 20% protection in children at 6 months after a single dose [8, 13, 14].

Children demonstrate robust vibriocidal responses to cholera vaccines but experience lower vaccine efficacy and a shorter duration of protection compared with adults [6–8]. For this reason, we hypothesized that the vibriocidal titer may not predict protection from infection in children as accurately as in adults. By prospectively observing a large cohort of children and adults exposed to *V cholerae* and closely following their clinical outcomes, we evaluated the utility of the vibriocidal titer as a marker of protection in different age groups. To include a larger number of young children, we combined 2 cohorts of household contacts that we previously described and added 1 new cohort to the analysis to enable evaluation of all age groups with the largest possible sample size. We found that the vibriocidal titer was uniformly associated with protection against *V cholerae* regardless of age.

The reasons for a lesser degree and shorter duration of protective immunity after vaccination in children may be due to fewer lifetime exposures to *V cholerae*, resulting in less immunologic priming upon re-exposure. In this model, exposure to *V cholerae* generates long-lived *V cholerae*-specific memory B cells that respond with differentiation and proliferation upon re-exposure to *V cholerae* antigens. Detectable circulating *V cholerae-*specific memory B-cell responses correlate with protection from infection [15]. Similar to the vibriocidal titer, memory B-cell responses to *V cholerae* increase with age in cholera-endemic areas, suggesting that responses develop after a lifetime of exposures to *V cholerae* antigens [4]. Our results suggest that the relationship between the vibriocidal titer and the ability of *V cholerae-*specific memory B cells to respond to re-exposure may differ between children and adults. In addition to lacking the immunologic priming of repeated exposures, this relationship between vibriocidal titers and protection may be influenced by other host factors that differentiate adults and children, including immunologic immaturity, higher levels of enteric enteropathy and malnutrition, differences in intestinal parasitic burdens, or differences in the gut microbiome, a newly recognized host factor that is important for the development of mucosal immune responses.

Despite the pooling of 3 cohorts, our study is still limited by the number of children under 5 years of age. This may have reduced our power to detect smaller potential differences between the immune responses in different age groups or a difference specific to very young children under 2 years of age.

## CONCLUSIONS

Overall, we found that the vibriocidal antibody titer predicts protection from *V cholerae* infection across the age spectrum including young children, older children, and adults. Because children have comparable vibriocidal responses to cholera vaccines but lessened and shortened protection compared with adults who receive cholera vaccines, it is possible that the vibriocidal titer may not be the optimal marker to determine protection afforded by cholera vaccines in young children. Improved markers of protective immunity are needed for measuring vaccine efficacy in children, especially very young children, who are vulnerable to cholera.
